# Infectious wrist arthritis complicating acute carpal tunnel syndrome in a child: A CARE-compliant case report

**DOI:** 10.1097/MD.0000000000039276

**Published:** 2024-08-16

**Authors:** Jihui Huang, Jun Li, Ruichen Li, Xing Wu, Yuanxue Lei, Zhiguo Zhou

**Affiliations:** a Medical College, Wuhan University of Science and Technology, Wuhan, China; b Wuhan Children’s Hospital (Wuhan Maternal and Child Healthcare Hospital), Tongji Medical College, Huazhong University of Science & Technology, Wuhan, China.

**Keywords:** acute carpal tunnel syndrome, case report, children, infectious wrist arthritis, vacuum sealing drainage

## Abstract

**Introduction:**

The objective of this case report is to provide clinical evidence that acute infectious wrist arthritis in children can lead to the rare condition of acute carpal tunnel syndrome (ACTS). This article discusses in detail the characteristics of infectious wrist arthritis complicating ACTS in children in terms of etiology, pathogenic bacteria, treatment modalities, and sequelae to improve the understanding of this disease.

**Patient concerns:**

A 10-year-old male child presented with a 15-day history of swelling and pain in the left forearm, wrist, and hand.

**Diagnoses:**

Left-sided infected wrist arthritis complicating ACTS.

**Interventions:**

The child received emergency surgery and anti-infective treatment combined with regular rehabilitation.

**Outcomes:**

During the treatment period, the child’s wrist pain and swelling gradually improved, and wrist movement was restored compared with the preoperative period. At 6-month follow-up, the activities of the metacarpophalangeal joints of the left hand were close to normal, and the flexion of the left wrist joint was slightly limited.

**Conclusion:**

In infectious wrist arthritis in children, ACTS is a serious complication that requires aggressive surgical carpal tunnel release to avoid median nerve injury in addition to anti-infective therapy.

## 1. Introduction

Infectious arthritis is a bacterial-induced intra-articular infection that accounts for about 21% of bone and joint infections in children, most commonly in the hip joint, followed by the knee joint, with the wrist joint belonging to the rare sites of development. Due to the unique tissue structure of the wrist, carpal tunnel tissue edema and a large amount of purulent secretion are produced during wrist infection, leading to compression of the median nerve, thus causing acute carpal tunnel syndrome (ACTS), which, if left untreated, can severely affect the function of the wrist. There are only 2 such cases in adults reported in the available literature.^[1,2]^ The author reviewed a case of infectious carpal arthritis complicating ACTS in a child diagnosed and treated in July 2023 and reported as follows.

## 2. Case presentation

### 2.1. Medical history

Child, male, 10 years 8 months. Hospitalized for “15 days of swelling and pain in the wrist and hand of the left forearm.” The child presented with swelling and pain in the wrist and hand of the left forearm 15 days ago without any obvious triggers, denying a history of trauma and insect bites. The swelling and pain gradually worsened, and the child was unable to move. He was seen in a local hospital 3 days ago and was treated with anti-infective therapy for 3 days without obvious recovery, and had a fever 2 days ago, with an unknown maximum temperature, and was referred to our hospital for further diagnosis and treatment.

### 2.2. Specialized physical examination

The left wrist and hand were markedly swollen and tender to palpation (+), with pain notable in the wrist and limited mobility; Tinel’s sign (+); wrist flexion test (+); the left side of the finger pulling pain was obvious; finger flexion, extension, adduction and abduction activities were limited, the radial side of the 3 and a half fingers had a sensory impairment, and the radial artery pulsation was weakened.

### 2.3. Imaging

**Figure 1.**
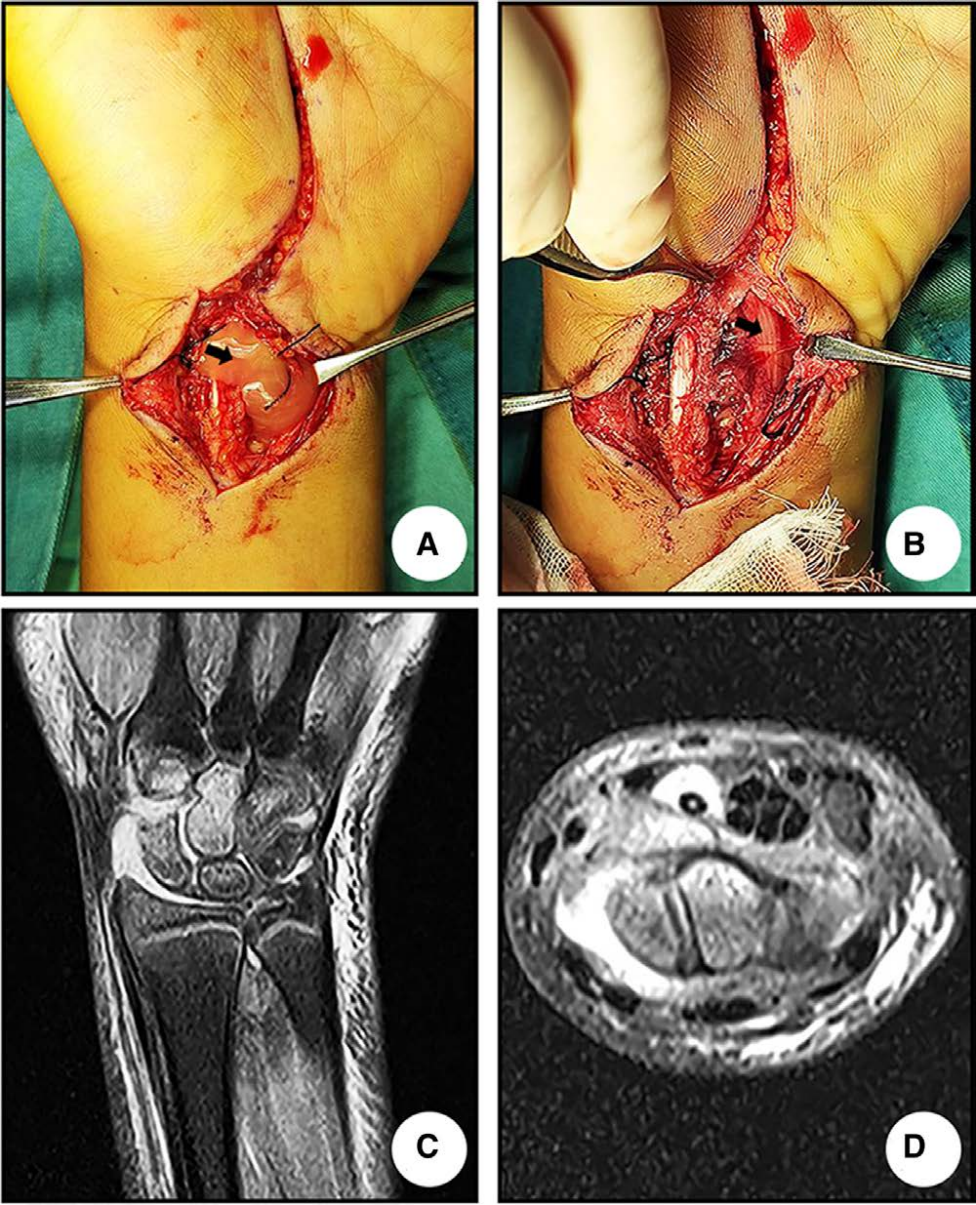
Surgical photo documentation and case imaging. (A) A large amount of yellow-brown pus overflow was seen between the radial carpal flexor and palmaris longus muscles. (B) Pale edematous median nerve. (C) Preoperative MRI coronal view of the left wrist showed bone marrow edema of the carpal bone with a large amount of high signal fluid around it. (D) Preoperative MRI axial view of the left wrist showed marked edema of the soft tissues in the carpal joint cavity, and effusion in the tendon sheath of the bunion spreader muscle and the carpal tunnel.

Preoperative magnetic resonance imaging(MRI) scanning of the left wrist joint showed: bone marrow edema of the bones of the left wrist joint; injury to the left anterior rotator anterior muscle, metacarpal muscle, contralateral spreader muscle, spreader muscle of the little finger, ligament of the periprosthetic wrist joint, deltoid fibrocartilaginous complex, and edema of the periarticular subcutaneous tissues; and accumulation of fluid in the cavity of the left carpal tunnel, carpal tunnel, and the tendon sheath of the spreader muscle of the longissimus dorsi muscle (Fig. 1C,D).

Preoperative X-ray examination suggested that the left ulnar radius was complete in shape and continuous in bone quality, with no obvious signs of fracture, and the left elbow joint gap was normal; the left forearm was swollen with soft tissue shadow.

### 2.4. Admission laboratory tests

White blood cell count: 9.45*10^9^/L, percentage of neutrophils: 75.7%, hypersensitive C-reactive protein: 48.80 mg/L, procalcitonin: 0.1 ng/ml, ESR: 31 mm/h. Anti-streptococcal hemolysin test, HLA-B27 test, serum antinuclear antibody complete set of 8 tests, whole blood T-cell test for tuberculosis infection (immunoblotting assay), whole blood bacterial culture identification, and drug sensitivity tests were all negative.

### 2.5. Diagnosis

The diagnosis of infectious wrist arthritis complicated by ACTS was made based on the patient’s history, physical examination, imaging, and laboratory findings.

### 2.6. Treatment

On the day of admission, he underwent emergency left wrist arthrotomy + carpal tunnel release + exploration of hand tendons, blood vessels, and nerves + closed negative pressure drainage of the wound (Fig. 1A,B). After surgery, the left wrist and hand were given negative pressure closed drainage, cefazolin sodium for anti-infection, and methylprednisolone sodium succinate for anti-inflammation (for 3 days).

## 3. Results

**Figure 2.**
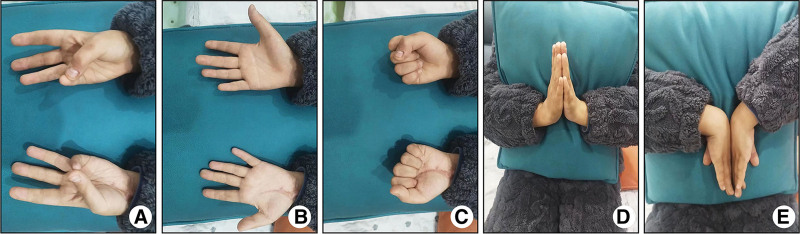
The child’s wrist mobility at 6 months postoperatively, with good recovery of hand function and 45°of wrist flexion on the left side, was worse than on the healthy side.

The child’s pus specimen bacterial culture was identified as Staphylococcus aureus. Postoperative review on day 7: white blood cell count, percentage of neutrophils, erythrocyte sedimentation rate, hypersensitive C-reactive protein, and procalcitonin were normal. At this time, the swelling of the left wrist subsided compared with the previous period, a slight voluntary movement of the fingers was visible, and the terminal blood flow of the finger ends was good. The child was treated with anti-infection and continuous vacuum sealing drainage after the operation, and the inflammatory indexes decreased significantly. The finger and joint activities were better than before, and the effect of the current treatment modality was obvious. Replacement of the vacuum sealing drainage (VSD) was performed on postoperative day 9. On the 18th postoperative day, surgical exploration of the left wrist joint was performed without any obvious infected necrotic tissue, and the incision was closed with sutures. On the 20th day of admission, the bacterial culture of the secretion was identified as negative, and all the indicators of inflammation were normal on review. At this time, the child’s wrist flexion and extension activities were not limited, and there was slight pain when moving, but the pain was not intense. Flexion and extension of the left thumb and index finger were fine, dorsiflexion and extension were slightly limited, and pain was obvious when dorsiflexion and extension were excessive. Flexion and extension of the third, fourth, and fifth fingers were good. He was discharged from the hospital and continued to take oral antibiotics for 1 week after discharge and insisted on functional training of the left wrist. At 6-month follow-up, the activities of the metacarpophalangeal joints of the child’s left hand were close to normal, and wrist flexion of the left wrist joint was slightly limited (Fig. 2).

## 4. Discussion

Septic infections of bones and joints in children seriously affect their growth and development. Septic arthritis accounts for about 21% of all bone and joint infections in children, with the most common site of involvement being the hip joint in about 43% of cases, followed by the knee and elbow joints. Septic wrist arthritis in children is very rare. Li et al^[3]^ conducted a multicenter review of infectious arthritis of the upper extremities in children and found that only 4% of 684 patients had wrist infections. Strong et al^[4]^ reported 6 cases of infectious wrist arthritis in infants, and all of these 6 children had multi-joint infections, whereas this article reports that the child had a single wrist infection.^[5]^

The most common pathogen in infectious wrist arthritis is Staphylococcus aureus,^[6]^ followed by Streptococcus pyogenes and Streptococcus pneumoniae, and methicillin-resistant Staphylococcus aureus, which has the characteristics of strong resistance and high virulence, has shown a tendency to be detected at a higher rate in infections of the bone and joint in recent years.^[3]^ There are 3 main routes of invasion of the joints by pathogens of infectious wrist arthritis: hematogenous route, away from the lesion in the affected joint; diffusion of neighboring foci, such as osteomyelitis of the ulnar radial diaphysis to the epiphysis and wrist joint; direct contamination due to the special anatomical structure of the wrist when the palm is traumatized, pathogenic microorganisms can spread to the carpal tunnel through the mid-palm gap. In addition to this, there are a small number of medical infections, such as those caused by carpal puncture, carpal arthroscopy, and internal fixation of carpal fractures. In this article, we report a child with no open trauma to the left upper extremity, and in the light of the history, we consider a carpal infection caused by the hematogenous route.

Infectious wrist arthritis in children can lead to a variety of complications, with the destruction of cartilage starting in the eighth hour of infection, which can further spread to the subchondral bone and lead to osteomyelitis. Parnes et al^[7]^ reported a case of infectious wrist arthritis due to Streptococcus pyogenes in a child, who was found to present with a spontaneous total metacarpal fusion on long-term follow-up. If the infection occurs in the carpal tunnel it can cause ACTS, which was first reported in 1989 by Gerardi JA^[2]^ in a 23-year-old male patient, and later Yoshida et al^[8]^ summarized the characteristics of patients with ACTS due to infections, and only 1 out of 15 patients was a child. Carpal tunnel syndrome (CTS) is the most common peripheral nerve entrapment syndrome, which occurs mainly in adults and tends to have a chronic onset, whereas a rapid increase in intracarpal tunnel pressure due to infection, trauma, and coagulation disorders often causes ACTS.^[9]^ Because the carpal tunnel is a unique structure of the wrist, with tendons and the median nerve passing through it, and the tissue structure is tightly arranged and inelastic when an infection occurs in the wrist, it is easy to cause edema of the tissues in the carpal tunnel and produce a large amount of purulent secretion, which leads to a sharp rise in the pressure in the carpal tunnel, compression of the median nerve, and the formation of ACTS. In the early stage of X-ray examination, only soft tissue swelling in the wrist can be observed, and MRI examination is of great significance for diagnosis, which can determine the degree of spread of the lesion and help determine the surgical method. In this report, the preoperative MRI examination (Fig. 1C,D) showed that the soft tissues in the carpal tunnel were swollen and there was a large amount of fluid, which had pressed the median nerve, combined with the medical history and physical examination, this was an indication for emergency surgery.^[10]^ Blood tests provide evidence of infection, and of the inflammatory markers hypersensitive C-reactive protein takes the shortest time to return to normal and can be used as a dynamic test of treatment efficacy.

Infectious wrist arthritis in children should be treated with anti-infective therapy as early as possible after a clear diagnosis.^[11]^ Once ACTS symptoms such as numbness of the wrist, abnormal sensation, and weakness of the big fish are found, wrist puncture is feasible, and the diagnosis can be further clarified if pus is found out by puncture, and then surgical incision and decompression should be performed as early as possible, and adequate drainage becomes the key to saving the function of the wrist.^[12]^ The surgical incision site should be a Z-shaped incision on the palmar side of the wrist. Considering the children with increased pressure in the carpal tunnel, the transverse carpal ligament should be decisively incised to decompress, the tendon and median nerve should be carefully explored, and necrotic tissues should be removed. According to the imaging examination and the anatomical characteristics of the fascial space at the wrist, an additional incision can be made on the palmar side of the hand to explore the mid-palmar space.^[13]^ Choudhury et al^[14]^ used arthroscopic flushing of the carpal joint in adult infectious wrist arthritis and placed a catheter intraoperatively to facilitate continuous flushing of the joint cavity postoperatively, but the use of arthroscopy in pediatric carpal joints is limited due to the small space of the carpal joint. In this case, after adequate irrigation of the carpal joint cavity and release of the carpal tunnel, VSD was performed, and good therapeutic results were achieved. In recent years, VSD has been widely used as a simple and effective method in osteoarthritic infections,^[15]^ but there is less experience in the application of VSD in infectious wrist arthritis, and the exact efficacy needs to be further confirmed.

Evaluation of the efficacy of infectious wrist arthritis should not only focus on the elimination of infection but also on the recovery of joint function of the wrist after anti-infection and surgical treatment should be the key evaluation index. In this case, the child’s wrist sensation was restored, and there were no sequelae of median nerve injury after timely surgical treatment. At the same time, due to the large number of tendon structures in the carpal tunnel and the small space, the possibility of tendon adhesion was high, so the author considered that the child’s wrist flexion function was poorer than that of the healthy side because of the tendon adhesion caused by the inflammation (Fig. 2). Therefore, restoration of wrist function requires early postoperative rehabilitation training, and most scholars believe that the timing of rehabilitation training should begin 24 hours after surgery.^[12]^

### 4.1. Limitations of the study

Acute infectious wrist arthritis in children is very rare, and the pathogenesis needs further study. Second, preoperative carpal function pictures could not be provided due to the patient’s young age and uncooperative preoperative physical examination. In addition, this case describes the results of a 6-month postoperative follow-up, and the patient’s long-term outcome needs to be further tracked until skeletal maturity.

## 5. Conclusions

In conclusion, infectious wrist arthritis complicating ACTS as a rare case of osteoarticular infection in children can cause nerve and tendon damage, which in turn leads to the loss of wrist function. Aggressive surgery after early MRI diagnosis, together with anti-infection treatment, is an important means to save joint function, and early functional exercise is favorable for the recovery of wrist and hand function to avoid residual joint dysfunction.

## Acknowledgments

We are very grateful to the patient, the guardians, and all the researchers who supported us in this study. The authors have no funding and conflicts of interest to disclose.

## Author contributions

**Writing – original draft:** Jihui Huang

**Data curation:** Jun Li

**Formal analysis:** Ruichen Li

**Supervision:** Xing Wu

**Methodology:** Yuanxue Lei

**Writing – review & editing:** Zhiguo Zhou

## References

[1] Zbeda RM Rabinovich RV Vialonga M Seigerman DA. Acute septic carpal tunnel syndrome in a rock climber. J Orthop Case Rep. 2021;11:100–3. 34327176 10.13107/jocr.2021.v11.i04.2170 PMC8310637

[2] Gerardi JA Mack GR Lutz RB. Acute carpal tunnel syndrome secondary to septic arthritis of the wrist. J Am Osteopath Assoc. 1989;89:933–4. 2768010

[3] Li Y Sanborn RM Cook D. Children’s Orthopaedic Trauma and Infection Consortium for Evidence-Based Studies (CORTICES). Descriptive epidemiology of upper extremity septic arthritis in children-review of a retrospective multicenter database. J Pediatr Orthop. 2023;43:46–50. 36044373 10.1097/BPO.0000000000002266

[4] Strong M Lejman T Michno P. Septic arthritis of the wrist in infancy. J Pediatr Orthop. 1995;15:152–6. 7745084

[5] Silver JM Hennrikus W. Septic arthritis of the pediatric wrist: a case report and review of the literature. Cureus. 2020;12:e7444. 32351823 10.7759/cureus.7444 PMC7186098

[6] Low SL Jennings JD Clippinger BB Landfair GL Criner-Woozley KT Ilyas AM. Predictors of septic wrist: a dual-center 10-year review of risk factors. J Hand Microsurg. 2020;12:19–26. 32280177 10.1055/s-0039-1693068 PMC7141905

[7] Parnes N Green CK Scanaliato JP Caruso J Dunn JC. Spontaneous pan-carpal metacarpal fusion after a case of pediatric septic arthritis of the wrist: a case report. JBJS Case Connect. 2022;12:e22.00066. 10.2106/JBJS.CC.22.00066 35385412

[8] Yoshida H Imura H Goto T Nakamata T Daya MR Kamiya T. Acute carpal tunnel syndrome due to pyogenic flexor tenosynovitis without any antecedent injury. Intern Med. 2017;56:1439–42. 28566613 10.2169/internalmedicine.56.7584 PMC5498214

[9] Birman MV Strauch RJ. Management of the septic wrist. J Hand Surg. 2011;36:324–6; quiz 327. 10.1016/j.jhsa.2010.11.034 21276897

[10] Krauss S Denzinger M Rachunek K Kolbenschlag J Daigeler A Illg C. Septic arthritis of the wrist: a retrospective review of 39 cases. J Hand Surg Eur Vol. 2022;47:812–7. 35642094 10.1177/17531934221101805

[11] Dadras M Bohm C Wallner C. Long-term results of bacterial septic arthritis of the wrist. J Plast Reconstr Aesthet Surg. 2018;71:1138–45. 29891427 10.1016/j.bjps.2018.04.017

[12] Ku YC Gannon M Fang W Norcini RC Woodberry KM. Management of acute carpal tunnel syndrome: a systematic review. J Hand Surg Glob Online. 2023;5:606–11. 37790823 10.1016/j.jhsg.2023.06.012 PMC10543818

[13] Ahlawat S Corl FM LaPorte DM Fishman EK Fayad LM. MDCT of hand and wrist infections: emphasis on compartmental anatomy. Clin Radiol. 2017;72:338.e1–9. 10.1016/j.crad.2016.11.020 28065641

[14] Choudhury MM Jiang J Yap R. A technique of continuous catheter irrigation in an infected wrist joint: improving management in septic arthritis of the wrist. J Wrist Surg. 2023;12:549–57. 38213558 10.1055/s-0042-1749657 PMC10781570

[15] Yang R Hua H Wang X Guo Z Zhong W. Vacuum sealing drainage combined with eggshell-like debridement antibiotic-loaded calcium sulphate for calcaneal osteomyelitis. J Orthop Surg Res. 2023;18:796. 37875933 10.1186/s13018-023-04259-6 PMC10594815

